# IEDB‐3D 2.0: Structural data analysis within the Immune Epitope Database

**DOI:** 10.1002/pro.4605

**Published:** 2023-04-01

**Authors:** Marcus Mendes, Jarjapu Mahita, Nina Blazeska, Jason Greenbaum, Brendan Ha, Kelly Wheeler, Jiyao Wang, Deborah Shackelford, Alessandro Sette, Bjoern Peters

**Affiliations:** ^1^ Center for Infectious Disease and Vaccine Research La Jolla Institute for Immunology La Jolla California USA; ^2^ Leidos Company San Diego California USA; ^3^ National Center for Biotechnology Information National Library of Medicine, National Institutes of Health Bethesda Maryland USA; ^4^ Department of Medicine University of California, San Diego La Jolla California USA

**Keywords:** adaptive immunity, antigens, database, epitopes, immunoinformatics, structural biology

## Abstract

The Immune Epitope Database (IEDB) catalogs T cell, B cell, and major histocompatibility complex ligand information in the context of infectious disease, allergy, autoimmunity, and transplantation. An important component of this information is three‐dimensional structural data on T cell receptors, antibodies, and pairwise residue interactions between immune receptors and antigens, which we refer to as IEDB‐3D. Such data is highly valuable for mechanically understanding receptor:ligand interactions. Here, we present IEDB‐3D 2.0, which comprises a complete overhaul of how we obtain and present 3D structural data. A new 3D viewer experience that utilizes iCn3D has been implemented to replace outdated java‐based technology. In addition, we have designed a new epitope mapping system that matches each epitope available in the IEDB with its antigen structural data. Finally, immunogenicity data retrieved from the IEDB's ImmunomeBrowser can now be used to highlight immunogenic regions of an antigen directly in iCn3D. Overall, the IEDB‐3D 2.0 provides an updated tool platform to visualize epitope data cataloged in the IEDB.

## 1 INTRODUCTION

The Immune Epitope Database and Analysis Resource (IEDB) (Vita et al., 2018) is a freely available resource that contains an extensive collection of experimentally measured B cell, T cell, and major histocompatibility complex (MHC) ligand data for infectious diseases, allergens, autoimmune diseases, and transplant/alloantigens. The IEDB contains information from more than 66,000 antigens, which are proteins that trigger an immune response, available on the website.

In 2011, a comprehensive description of the components that formed the IEDB‐3D (Ponomarenko et al., 2011) was published. IEDB‐3D provides three‐dimensional structural data on curated information of lymphocyte T cell receptors (TCRs), B cell receptors/antibodies (BCRs), MHC molecules, and the epitopes to which they bind, as well as pairwise residue interactions between immune receptors and antigens. To visualize the intermolecular contacts and interface areas, the IEDB implemented the EpitopeViewer, a web browser‐based Java application that was able to handle all curated structural data within the IEDB (Beaver et al., 2007).

Over the last decade, many new approaches and tools have become available in structural biology. We have also received many requests from IEDB users regarding how they would like to see data visualized on 3D protein structures. This has identified several major areas for improvement, as described below.

### 1.1 3D EpitopeViewer

At the time of implementation in 2007, the EpitopeViewer housed impressive features, such as a 2D plot of interactions between epitope and receptor residues, color‐coding for each type of molecule preserved between all windows, and 3D visualization of curated interactions between epitope and receptor. However, over time, the Java plug‐in stopped being supported on commonly used browsers, which prompted the IEDB to replace the EpitopeViewer with the JSmol Molecule Viewer (Steinbeck et al., 2003). Unfortunately, JSmol does not possess the same features as the EpitopeViewer, and the graphical rendering has lower quality compared to other software, such as ChimeraX (Goddard et al., 2018), iCn3D (Wang et al., 2022), Pymol (Schrodinger, 2015), or Mol* Viewer (Sehnal et al., 2021). We thus wanted to implement a new epitope viewer in the IEDB that takes advantage of modern protein visualization tools and provides users with features specific to the epitope data contained in the IEDB.

### 1.2 3D models of antigens

The vast majority of epitopes cataloged in the IEDB are not derived from 3D structure data but rather by high throughput approaches, such as testing peptides for recognition by antibodies. Users want to see where such epitopes are located in the context of their source antigen. In the past, the IEDB provided tools to assist in creating homology models of proteins and mapping epitopes into them. However, these proved cumbersome for a casual user, and expert users would trust their models over those they could generate with IEDB tools. With the availability of precomputed 3D models for many proteins through Alphafold (Jumper et al., 2021), we could address these issues and make direct visualizations of many epitopes available for users not interested in creating their own homology models.

### 1.3 3D visualization of immunogenicity

A prominent feature in the IEDB is the ImmunomeBrowser tool (Dhanda et al., 2018), which maps epitope recognition information back to an antigen, and computes an immunogenicity score for each position in that antigen. This can identify immunogenic hotspots of epitope recognition in a protein as compared to other areas that are not recognized. These data have so far only been plotted based on the linear sequence of the proteins. We wanted to also provide a 3D visualization of these data to show how immunogenic regions in a protein colocate in 3D space.

To address these main areas of improvement, we have substantially revised all components of the IEDB regarding structural data. We refer to this collection of coordinated updates as IEDB‐3D 2.0, which we describe in detail in Section 2.

## 2 RESULTS

**FIGURE 1:**
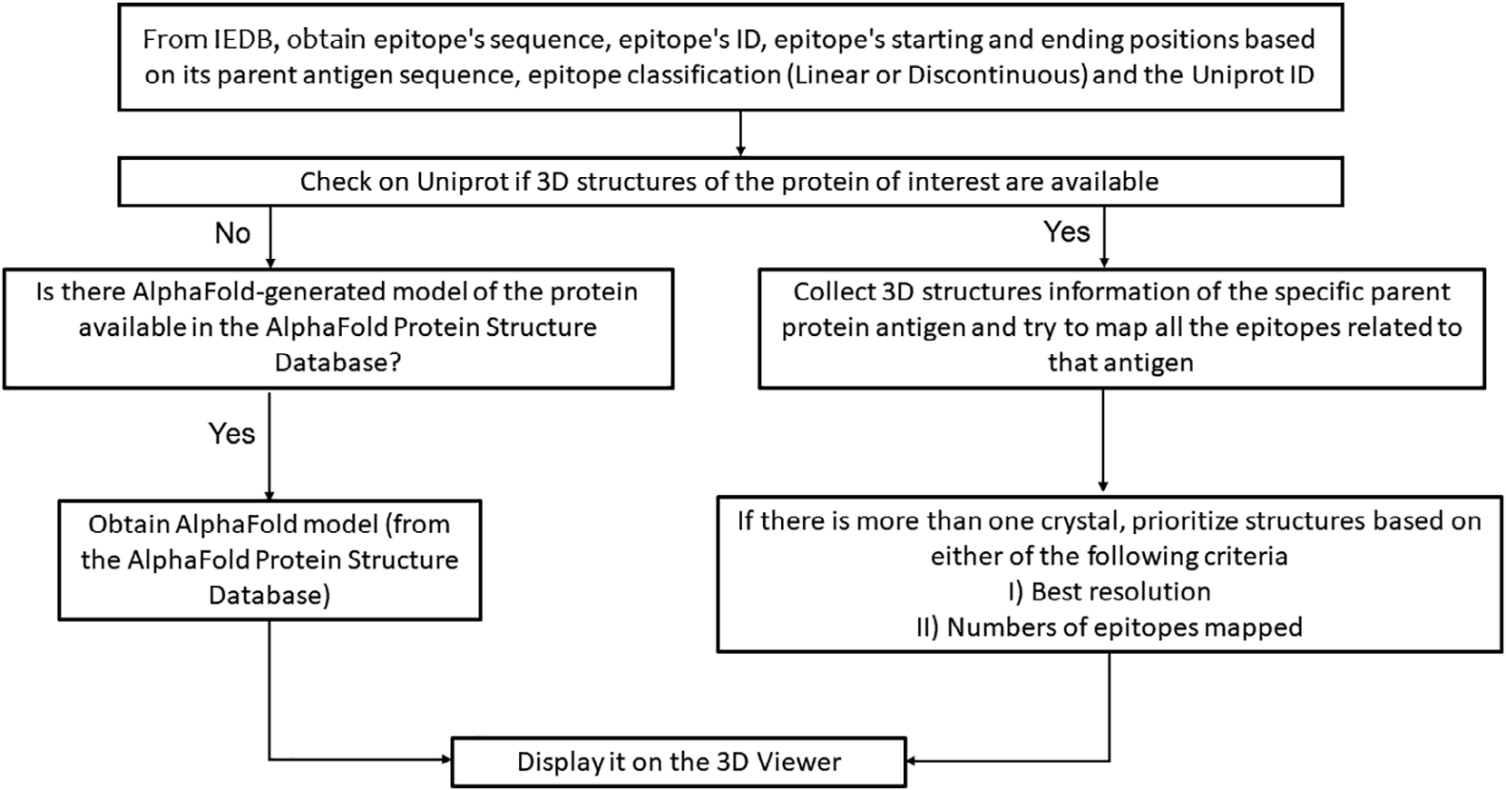
Flowchart showing the steps to map epitopes into antigens' structural data and display it on iCn3D. If the antigen does not have any atomic model or AlphaFold‐modeled available, it will not be included on our mapping system. The script regularly checks for new 3D structure releases.

IEDB‐3D 2.0 brings some modifications to the IEDB website. The following sections of the article describe the mapping of epitopes to the 3D structures of antigens and how to access it on the IEDB webpage (www.iedb.org). Figure 1 brings an overview of the mapping process in the form of a flowchart. If an antigen does not contain any structural data, no atomic model will be displayed for that protein. We recurrently check for new 3D structural data of antigens and will include them in our epitope mapping approach when available.

### 2.1 Mapping epitopes onto the structure of their source antigens

Mapping epitopes onto their corresponding 3D antigen structures required us to overcome several challenges. For some proteins, several structures of the same protein, each with varying length and resolution, are present in the Protein Data Bank (PDB). Choosing a single, representative structure from this pool can be problematic. Structures of flexible regions such as loops or intrinsically disordered regions are difficult to solve by experimental techniques such as X‐ray crystallography, cryo‐electron microscopy, and nuclear magnetic resonance (NMR) spectroscopy. Dealing with these unmodeled regions, nonlinear epitopes, and the difference between the residue positions in the protein sequence obtained from UniProt versus their positions in the structures retrieved from the PDB, made this process quite challenging. The problem is further compounded by the presence of more than 1 million epitopes in the IEDB, scaling the complexity. Some solutions are described in the literature to map the residues from the protein sequence onto its related atomic model (Dana et al., 2019; Faezov and Dunbrack, 2021). Nevertheless, none have been applied to map epitopes on structures of antigens.

Every epitope available in the IEDB has a unique ID. For our tool, the first step of the mapping process is to get the epitope sequence, ID, parent antigen UniProt ID, position in its parent antigen sequence, and classification (linear or discontinuous). Briefly, parent antigens are generally proteins from the UniProt reference proteome that are most closely related to the source protein of an epitope.

Using the UniProt ID of the parent antigen, the script compiles a list of PDB IDs related to that protein using the UniProt API. We also obtain the length and resolution of each PDB structure related to the antigen, which is available on the UniProt database. If there is no experimental structure available for that protein, the UniProt ID is included in a separate list, which is used to search the AlphaFold database, described in a later section.

The next step is to map the epitope residues onto the structures of the parent antigens. In order to achieve that, it uses data from the Structure Integration with Function, Taxonomy, and Sequence (SIFTS) (Dana et al., 2019), which is a project in the PDBe‐KB resource for residue‐level mapping between UniProt and PDB entries. Using the SIFTS API, an eXtensible Markup Language (XML) file containing each residue's position in the PDB file and corresponding UniProt sequence is downloaded. The epitope is mapped onto each chain of the antigen atomic model by combining the data from the SIFTS XML file, and the epitope description pulled from the IEDB.

The script generates several outputs per parent antigen. An epitope‐based output contains information on mapped epitopes, including epitope ID, PDB ID, residue position number and chain identifier, and the one‐letter code of the residues. A second, PDB‐centric output includes a list of PDB IDs to which epitopes have been mapped. For each PDB ID, the file includes the resolution and length of the structure and the number of epitopes mapped. To reduce the number of structures presented to users, the structures are prioritized according to their resolution as well as the number of epitopes mapped to them. We set a 20% identity cutoff for the epitope sequences. After attempting to map the entire epitope, if less than 20% of the residues are mapped onto the structure, we exclude the epitope from the output files. The 20% identity cutoff is given to avoid an epitope being mapped into a 3D structure that shares a low identity between them, which is noninformative and could lead to misinterpretations. The final output is a JSON file that contains the list of all UniProt IDs related to proteins that do not have any experimental structures available.

In order to map epitopes on antigens with no experimentally‐determined structure available, we leveraged the AlphaFold Protein Structure Database (Varadi et al., 2022). The database contains modeled structures of proteins present in different species such as *Homo sapiens* (human), *Mus musculus* (mouse), *Drosophila melanogaster* (fruit fly), and *Danio rerio* (zebrafish), to name a few. These structures were modeled using AlphaFold (Jumper et al., 2021), a deep learning method developed to predict structures of proteins based on their sequences. The software achieved the highest rank in the 14th Critical Assessment of Protein Structure Prediction challenge, demonstrating its power.

During the process of retrieving experimentally‐derived structures of antigens from the PDB for mapping epitopes, we generate a list of UniProt IDs as a JSON file for (i) antigens lacking experimental structures and (ii) antigens with available atomic models but with no epitopes mapped due the 20% identity cutoff. This JSON output is fed into another script, which retrieves all available AlphaFold models from the AlphaFold Database corresponding to the UniProt IDs. The linear and discontinuous epitopes are then mapped to the AlphaFold models of the relevant protein antigens employing a strategy similar to that used for mapping epitopes onto experimental structures. However, this process is less complex as the sequence used in the AlphaFold model is the same as the UniProt sequence for a given protein, thus avoiding the complications of having different position numbers for the same residue within a protein.

### 2.2 Displaying structural data from IEDB in a 3D viewer

The earlier version of IEDB‐3D utilized EpitopeViewer, a 3D viewer tool, to render and enable visualization of curated structural data present in the IEDB. EpitopeViewer was designed to work with curated data by using an XML file created by a structural curator. This file contains information such as residue contacts between adaptive immune receptors or MHC molecules and their epitopes and calculated interactions, such as electrostatic contacts, salt bridges, and hydrogen bonds.

iCn3D (Wang et al., 2022) is a 3D viewer developed by the National Center for Biotechnology Information that allows practitioners from different levels of expertise to (i) analyze protein–protein, protein–antigen, and mutation analysis interactions, (ii) calculate and extract contact maps, (iii) compute and inspect electrostatic potential calculated by Delphi program on‐the‐fly, pictured in the Figure S1, (iv) easily calculate a dynamic symmetry, (v) open several atomic models at once and make a multiple chain alignment, and realignment, and (vi) to visualize either all chains (asymmetric unit) or only the biological unit.

**FIGURE 2:**
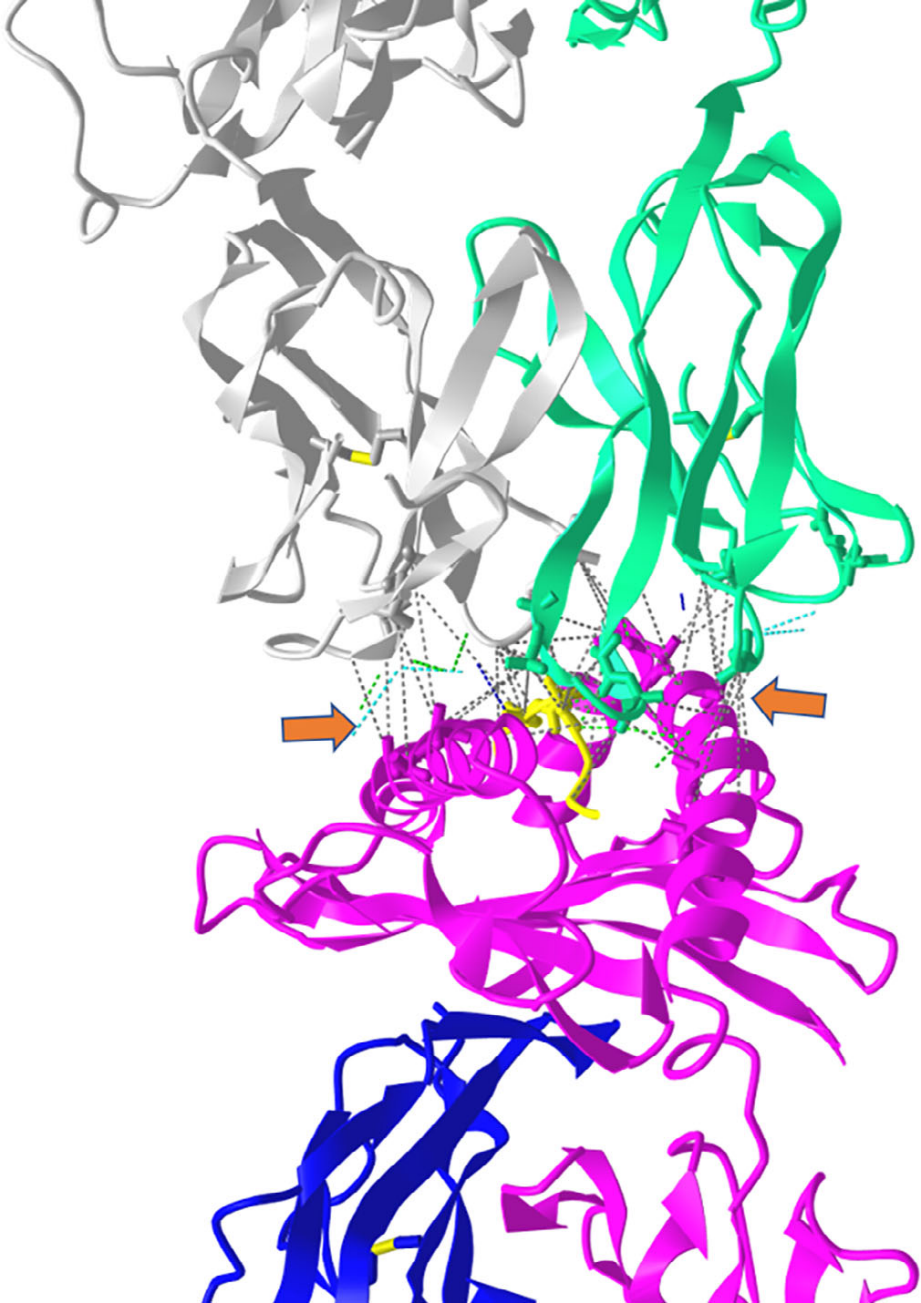
Using a TCR/pMHC complex (PDB ID: 1LP9) as input, iCn3D can easily calculate the non‐covalent bond's interaction between the alpha (white) and beta (green) chain of the TCR with alpha regions 1 and 2 of the MHC (pink) and peptide (yellow) inside the MHC cleft. Cysteine residues are automatically colored yellow. The dotted lines show the interactions, distinguished by the color of the dots. Green dots show hydrogen bonds, light blue dots are salt bridge/ionic, and gray are any other type of contacts/interaction. The orange arrows help to illustrate the interaction regions. The user can also generate a map of interaction in a separate window (figure not shown). MHC, major histocompatibility complex; TCR, T cell receptor.

iCn3D can calculate the interaction between the epitope and the receptor on the fly. It can calculate six interactions between molecular structures: contact, hydrogen bond, salt bridge or ionic interaction, halogen bond, π‐cation interaction, and π‐stacking. Also, the user can plot a map of those interactions and export it as a figure. Figure 2 illustrates how iCn3D displays an interaction between TCR and MHC loaded with an epitope.

**FIGURE 3:**
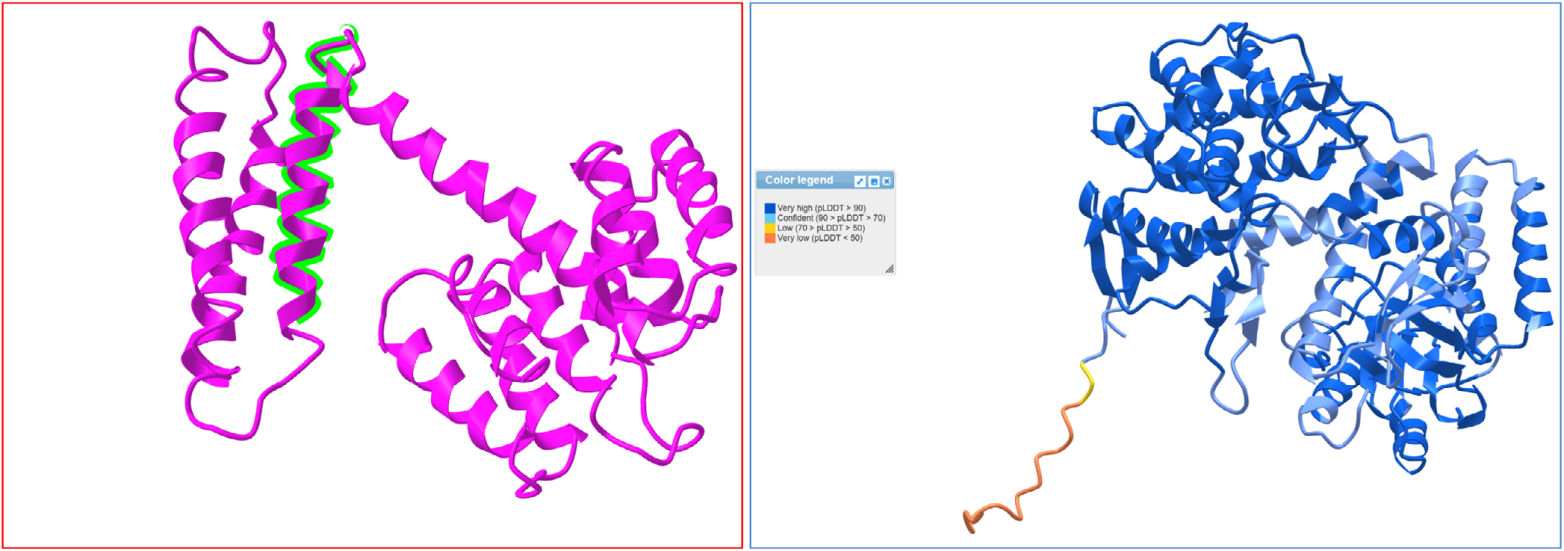
The figure shows an influenza A virus epitope (ID: 65389) mapped into a Matrix protein 1 antigen (PDB: 7JM3) on the left (red box) and an AlphaFold‐modeled Chaperonin GroEL 2 (AlphaFold ID: AF‐P09239‐F1) from Mycobacterium leprae, on the right (blue box). Both structures are displayed on iCn3D. The colors on the AlphaFold‐modeled protein indicate the confidence score (pLDDT), as shown in the legend box. The higher confidence suggests that the region is expected to be modeled well.

Another feature of iCn3D is the way it handles modeled proteins from AlphaFold. It automatically displays a table listing the pLDDT scores (confidence scores) labeled by color and shows how high each region's confidence is. A higher pLDDT score indicates that the region is modeled with high confidence. Figure 3 shows an example of a protein from AlphaFold displayed in iCn3D. NMR structures are also displayed on iCn3D, even if there is more than one conformer for that protein. In this case, the tool renders only one of the conformers, therefore the output is similar to any other type of 3D structure.

Initially, IEDB‐3D provided experimentally determined structural data of only TCR‐pMHC, pMHC, or antigen–antibody complex structures. With our new mapping system, IEDB‐3D 2.0 displays each epitope within the IEDB, in the context of the 3D structure of its parent antigen. To access the epitope viewer, we have a 3D icon in the “Epitopes” results tab of the IEDB. Figure 3 shows an example of an epitope mapped into an antigen displayed in iCn3D. If the same epitope is mapped into multiple parent proteins, all of the different antigens can be accessed by the dropdown menu available on the viewer. It is important to note that the “Epitopes” tab does not contain immunogenicity data. Users must access the ImmunomeBrowser Viewer if they are interested in assessing immunogenic regions of their structure. As a result, the epitope viewer only displays the epitope in the context of its antigen, being highlighted within the atomic model.

**FIGURE 4:**
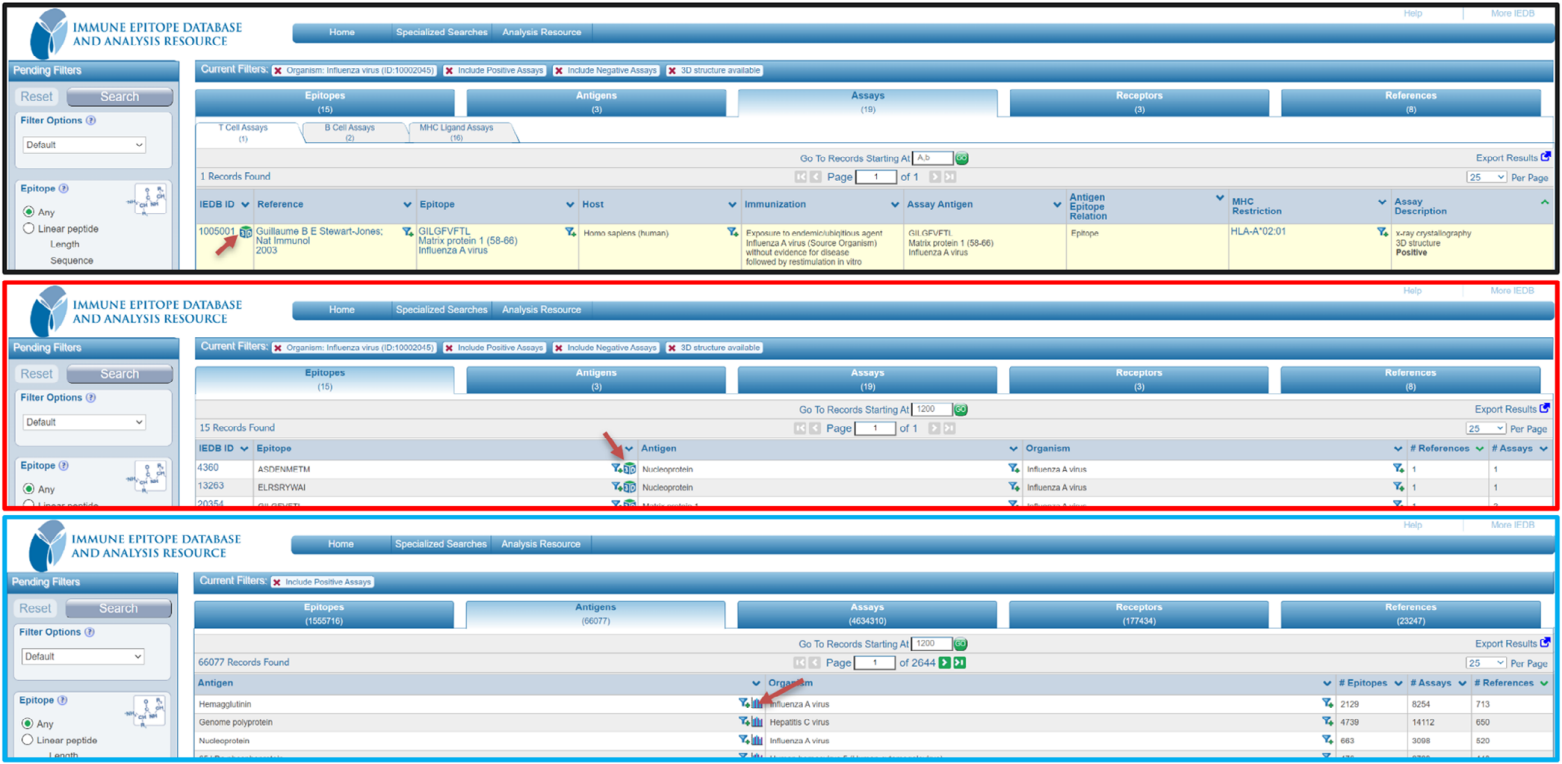
A print screen of three sections from IEDB where the user can access 3D information (highlighted with red arrows). For this example, we searched for the influenza virus on the IEDB's main page. The first section of the figure (on the top, with a black border) shows where the 3D icon appears when accessing via the “Assays” results tab for the experimentally‐determined structures of T cell, B cell, and MHC ligand assays. The second section (in the middle, with a red border) is related to the “Epitopes” tab. Suppose the epitope is mapped to a parent protein with an available experimentally‐determined crystal or a modeled protein from AlphaFold. In that case, there is a 3D icon in the “Epitope” column, which is used to visualize that epitope mapped in iCn3D. The third image (on the bottom, with a blue border) shows the ImmunomeBrowser button at the Antigens tab to access the IB page from that specific antigen. If that protein has any atomic model available, there will be an option on the new page to load the structure. IEDB, Immune Epitope Database; MHC, major histocompatibility complex.

We also maintained the previously available 3D icon in the “Assays” results tab to access structural information of lymphocyte TCRs, BCRs, and MHC molecules. This icon is a link to the iCn3D, which automatically loads the structure of the antigen with a user‐specified epitope mapped onto it. Figure 4 shows where the user can access the 3D icon. Lastly, iCn3D can also be accessed on the ImmunomeBrowser page, which can be accessed from the “Antigens” tab, and loads the parent protein antigen structural data. From here, researchers can select specific epitopes from the provided table to be displayed on the structure for further analysis.

### 2.3 Displaying immunogenicity data on the 3D structure of an antigen

The ImmunomeBrowser (Dhanda et al., 2018) is a tool that retrieves all epitopes available in the IEDB related to a given parent protein and calculates a score called the response frequency (RF). This score attempts to draw attention to regions of the antigen that are more immunogenic. The RF score is based on the number of positive assays at each position of the protein and uses the lower bound of the 95% confidence interval to provide a conservative estimate.

IEDB‐3D 2.0 integrates the RF score from the ImmunomeBrowser with the iCn3D viewer. The “Antigens” tab of the IEDB allows practitioners to access the ImmunomeBrowser by initially selecting an antigen to display in the browser. If the antigen's search process includes a filter with only MHC ligand assay data, the RF score for the entire antigen will be 0.0. It is necessary to contain T cell, B cell, or both assay data to avoid that situation.

From there, a placeholder window on the right of the page contains a drop‐down menu with a preselected list of up to five structure files. If there are more than five structures available for that antigen, the atomic models are first filtered by the number of epitopes mapped and the resolution of the structures, showing only those with the highest number of mapped epitopes or best resolution. At the bottom of the ImmunomeBrowser page, there is a table containing all epitopes available in IEDB for that specific antigen.

**FIGURE 5:**
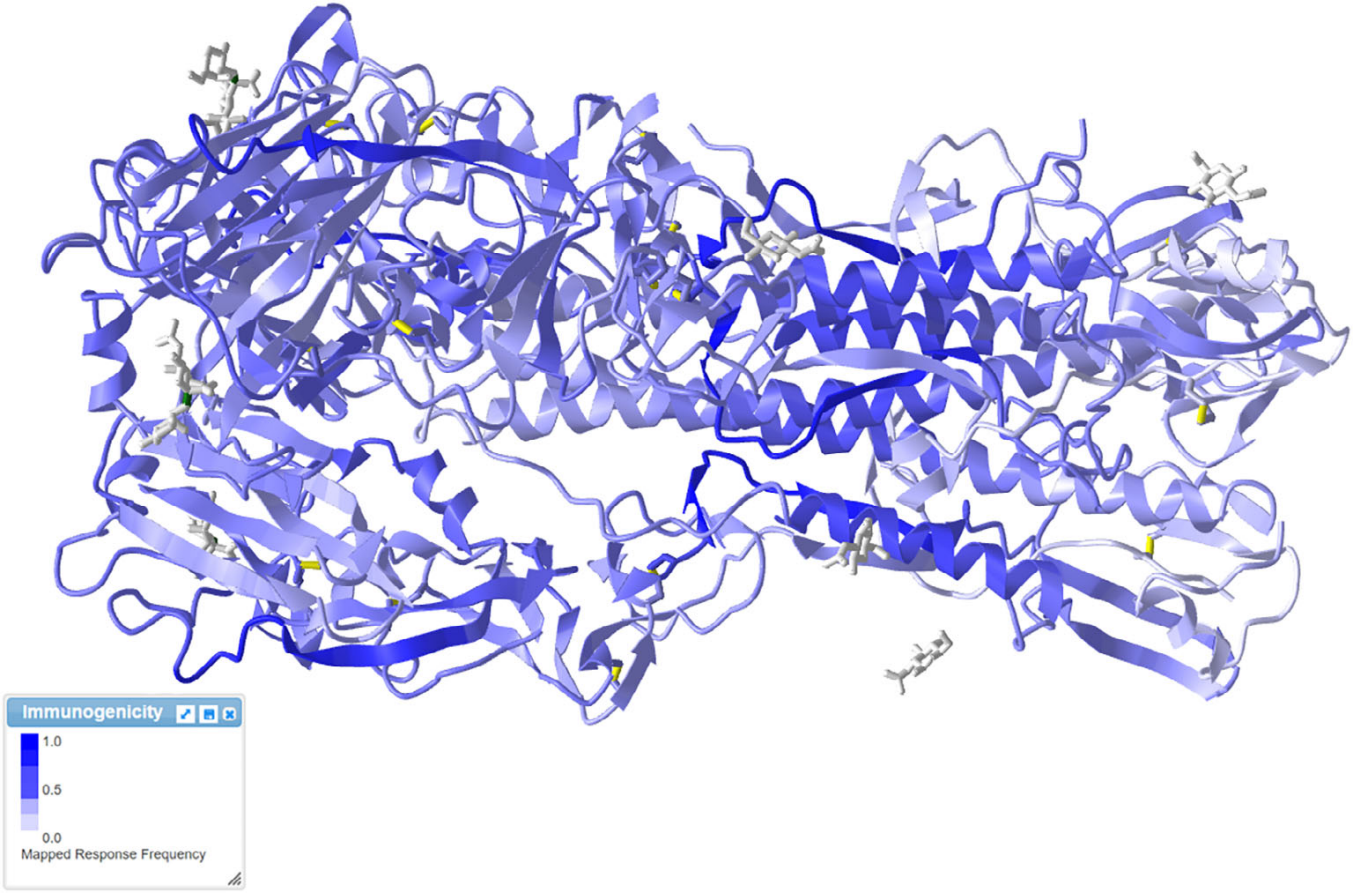
The hemagglutinin (PDB ID: 1RVX) from the Influenza A virus is colored based on the response frequency value from the ImmunomeBrowser. Regions in dark blue have more impact on the immune system's recognition, having a higher response frequency score. The light blue area indicates lower immunogenicity, with lower response frequency scores, and the white area shows regions with no immunogenicity. The immunogenicity color box contains the information needed to understand the colors.

Once the user chooses the high‐resolution atomic model to be displayed, the rows of the epitope table will change color to yellow, whether the epitope is mapped to the current structure or gray if the epitope is not included in the displayed 3D structure. The user can select any epitope from the table, which automatically highlights that epitope on the crystal structure and changes the color of the row to green. This selected atomic model's region is colored based on the RF score of the epitope. High RF scores will color the residue dark blue. In contrast, a low RF score will color the residue light blue, and regions with no immunogenicity will be colored white, as the user can check the immunogenicity color box. Figure 5 shows an example of the hemagglutinin antigen colored based on the RF.

### 2.4 Benchmark and metrics of the IEDB‐3D 2.0 content

The IEDB has a vast amount of data. As of August 2022, there were 1,730,172 peptidic epitopes available on the ImmunomeBrowser section in IEDB. From that number, we have 1,119,684 epitopes mapped on structural complexes and 610,488 epitopes not mapped. The mapped epitopes are split into 501,491 mapped on a PDB complex and 618,193 mapped on an Alphafold modeled structure.

There are various reasons as to why one‐third of the epitopes have not been mapped into atomic models. The first reason is that the antigen does not have an available 3D structure (neither crystals, NMR nor AlphaFold modeled). Second, some 3D protein structures contain regions that cannot be modeled. If the epitope falls in such a region, that atomic model is not included for that epitope, reducing the number of epitopes mapped. Third, mutated epitopes that have less than 20% of identity between the 3D structures and epitopes that are not mapped into any source protein, are also removed. Other issues are related to non‐mapping problems, including missing information on UniProt or the difference between the UniProt ID from IEDB and UniProt ID included in the SIFTS file.

Running the script on our server takes around 46 h to finish the mapping process using all the epitope data as an input for the first run. When rerunning it, we keep all the previously downloaded XML files and only remap the new epitopes, therefore taking less time. We renew the output quarterly, removing the previous results, and generating it again.

## 3 DISCUSSION

The amount of high‐resolution structure data is increasing every year. Linear sequence information is easier and faster to work with, but structural data brings us some insights that we can only achieve when considering the 3D conformation of proteins. With the greater amount of data available and the development of new open source tools such as AlphaFold to model antigens and iCn3D to display 3D structures on the browser, an update to IEDB‐3D was necessary.

In the new IEDB‐3D 2.0, our novel epitope mapping system works in conjunction with IEDB tools such as the ImmunomeBrowser, and external resources, such as iCn3D and the AlphaFold protein structure prediction tool. Now, any immunologist or structural biologist can easily access useful information, such as immunogenic regions in a specific antigen and how many non‐covalent interactions are occurring between a lymphocyte T cell receptor and an MHC docked with an epitope. They will also be able to display an electrostatic surface and check for the dissimilarity between a wild‐type crystal and a mutated protein.

Even with the increase in crystal numbers generated by X‐ray crystallography and Cryo‐EM combined with the structural proteins modeled using AlphaFold, we still do not have an antigen for each epitope available in the IEDB. Also, epitopes with modified residues (e.g., phosphorylation and citrullination) are removed from our mapping, even if they are highly immunogenic, due to incompatibility with our approach. So, unfortunately, there are plenty of non‐mapped epitopes. We expect an increase in the number of 3D structural data, especially with the increase in AlphaFold usage, and will, therefore, develop a secondary approach to include the modified residues and antigens that lack 3D information on UniProt.

There is a plethora of opportunities in how the IEDB‐3D 2.0 system can aid researchers across different fields of study, including immunotherapy against cancer, and rational drug and vaccine design for diseases.

## AUTHOR CONTRIBUTIONS

**Marcus Fabiano De Almeida Mendes:** Conceptualization (equal); investigation (equal); methodology (equal); software (equal); validation (equal); visualization (equal); writing – original draft (lead); writing – review and editing (lead). **Jarjapu Mahita:** Conceptualization (equal); data curation (equal); methodology (equal); software (equal); visualization (equal); writing – original draft (equal); writing – review and editing (equal). **Nina Blazeska:** Project administration (equal); supervision (equal); validation (equal); writing – review and editing (equal). **Jason Greenbaum:** Project administration (equal); software (equal); validation (equal); visualization (equal); writing – review and editing (equal). **Brendan Ha:** Data curation (equal); software (equal); visualization (equal). **Daniel Kelly Wheeler:** Data curation (equal); project administration (equal); software (equal); validation (equal); visualization (equal); writing – review and editing (equal). **Jiyao Wang:** Methodology (equal); software (equal); visualization (equal); writing – review and editing (supporting). **Deborah Shackelford:** Data curation (equal); investigation (supporting); writing – review and editing (supporting). **Alessandro Sette:** Funding acquisition (equal); investigation (supporting); methodology (supporting); supervision (supporting); writing – review and editing (supporting). **Bjoern Peters:** Conceptualization (equal); formal analysis (equal); funding acquisition (lead); investigation (equal); project administration (lead); resources (equal); supervision (equal); writing – review and editing (equal).

## Supporting information


**Figure S1.** The top image shows when the practitioner selects Delphi potential, available in the Analysis menu from iCn3D. A pMHC molecule is loaded, with the epitope displayed in brown and the alpha chain from the MHC in pink. The pop‐up box includes parameters that the user can change for the calculation. On the bottom image, we have the result from the Delphi calculation, displaying the surface with potential electrostatic. Electrostatic surface potentials are colored red and blue for negative and positive charges, respectively, and white represents neutral residues.

## Data Availability

The data that support the findings of this study are openly available at https://www.iedb.org.
