# Supplementary figures and images for: IEDB‐3D 2.0: Structural data analysis within the Immune Epitope Database
Source: Protein Sci. 2023 Apr 1;32(4):e4605. doi: 10.1002/pro.4605 (PMC10022491; doi:10.1002/pro.4605)

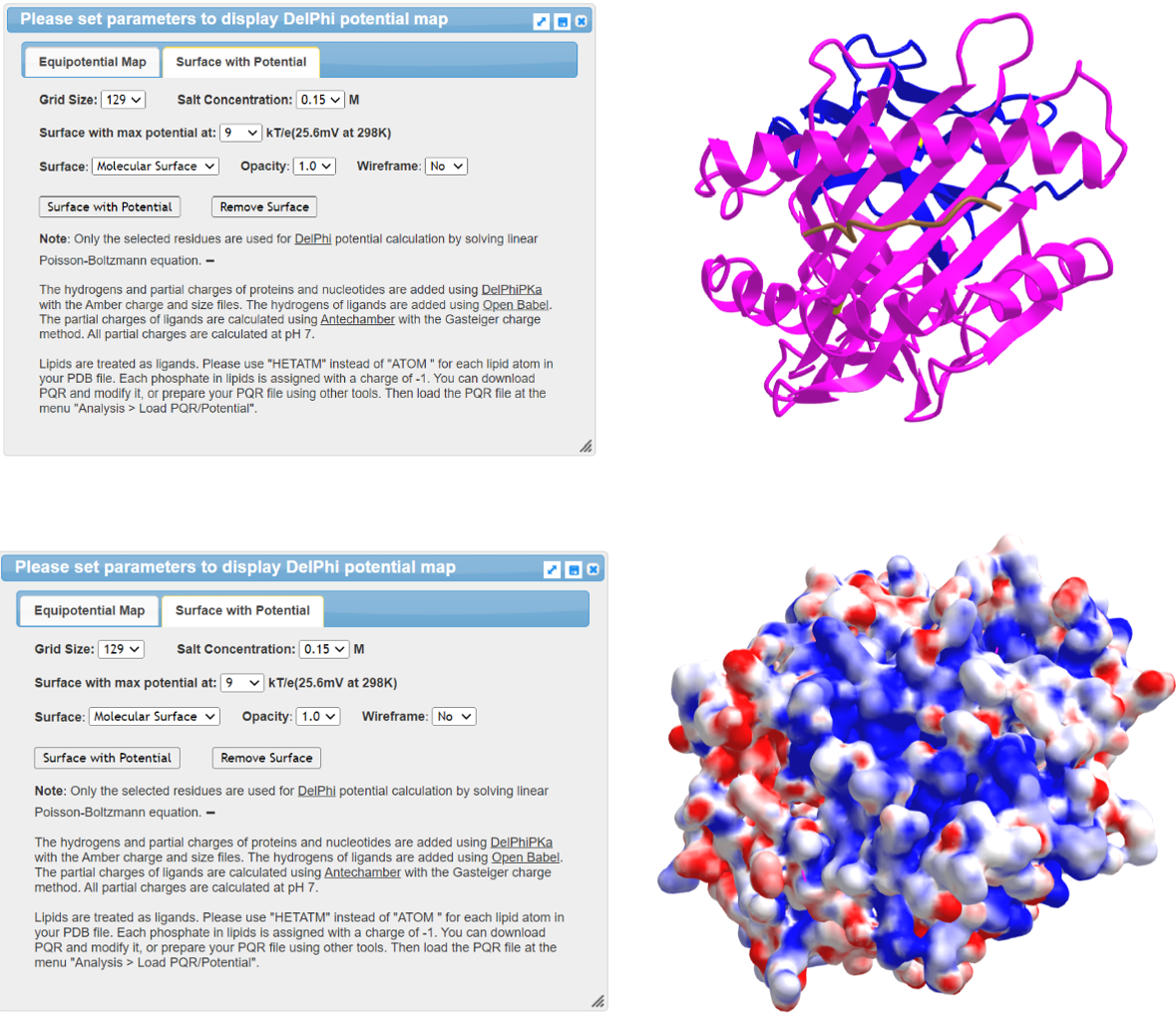

Supplement: Supplementary file 1 — Figure S1. The top image shows when the practitioner selects Delphi potential, available in the Analysis menu from iCn3D. A pMHC molecule is loaded, with the epitope displayed in brown and the alpha chain from the MHC in pink. The pop‐up box includes parameters that the user can change for the calculation. On the bottom image, we have the result from the Delphi calculation, displaying the surface with potential electrostatic. Electrostatic surface potentials are colored red and blue for negative and positive charges, respectively, and white represents neutral residues. [file PRO-32-e4605-s001.tif]
